# Next-Generation Sequencing of a Single Domain Antibody Repertoire Reveals Quality of Phage Display Selected Candidates

**DOI:** 10.1371/journal.pone.0149393

**Published:** 2016-02-19

**Authors:** Kendrick B. Turner, Jennifer Naciri, Jinny L. Liu, George P. Anderson, Ellen R. Goldman, Dan Zabetakis

**Affiliations:** 1 American Society for Engineering Education, Postdoctoral Fellow at the US Naval Research Laboratory, Washington, DC, United States of America; 2 American Society for Engineering Education, Science and Engineering Apprenticeship Participant at US Naval Research Laboratory, Washington, DC, United States of America; 3 Center for Bio/molecular Science and Engineering, US Naval Research Laboratory, Washington, DC, United States of America; National Cancer Institute, NIH, UNITED STATES

## Abstract

Next-Generation Sequencing and bioinformatics are powerful tools for analyzing the large number of DNA sequences present in an immune library. In this work, we constructed a cDNA library of single domain antibodies from a llama immunized with staphylococcal enterotoxin B. The resulting library was sequenced, resulting in approximately 8.5 million sequences with 5.4 million representing intact, useful sequences. The sequenced library was interrogated using sequences of known SEB-binding single domain antibodies from the library obtained through phage display panning methods in a previous study. New antibodies were identified, produced, and characterized, and were shown to have affinities and melting temperatures comparable to those obtained by traditional panning methods. This demonstrates the utility of using NGS as a complementary tool to phage-displayed biopanning as a means for rapidly obtaining additional antibodies from an immune library. It also shows that phage display, using a library of high diversity, is able to select high quality antibodies even when they are low in frequency.

## Introduction

The rise of Next-Generation Sequencing (NGS) technologies, which consist of a variety of related non-Sanger, high-throughput sequencing methods, over the past decade have had a profound effect across the field of biotechnology. Advances in NGS have enabled rapid, affordable genome sequencing [1], RNA quantification (RNA-seq) [2, 3], protein-DNA interaction determination (ChIP-seq) [4], and protein-RNA interaction determination (CLIP-seq) [5]. In contrast to traditional Sanger-based sequencing methods, NGS has the ability to evaluate millions of sequences in parallel, resulting in a more complete interrogation of the library in question. This capability makes NGS uniquely suited for characterization of an immune repertoire [6, 7]. Indeed, the technology has been successfully applied to human and zebrafish examples [8–11].

Recent new techniques for the application of NGS to antibody repertoires include a full pipeline for the isolation of functional antibodies that uses a DNA sequence database to construct a peptide library for comparison to liquid chromatography-mass spectroscopy data [12]. This allows for the direct identification of affinity purified antibodies without the construction of an expression library. Unfortunately this requires significant additional proteomics equipment. A novel method of molecular bar coding of cDNA sequences has also been proposed to help reduce sequencing 'noise' introduced by PCR [13]. In spite of the great promise of NGS, attention has been drawn to the difficulty of interpreting a diverse sequence database when there are artifacts introduced by PCR and sequencing errors [14].

Single-domain antibodies (sdAbs) are antibody fragments derived from heavy-chain-only antibodies found in camelids and possess a suite of desirable properties affording them unique advantages over conventional immunoreagents. These advantages include greater thermal stability, an ability to refold and maintain binding activity upon chemical or thermal denaturation, ease of engineering and production in *Escherichia coli* expression systems, and the capability of binding cryptic or buried epitopes [15–18].

The typical workflow for generating sdAbs begins with immunization of a camelid with an antigen, purification of mRNA from lymphocytes after an immune response has occurred, production of a cDNA library, construction of a phage-display library composed of the variable region of the heavy-chain antibodies, screening the library for binding phage, and characterization of the identified antibodies by DNA sequencing. The functional sdAbs isolated through this process represent only a fraction of those potentially present in the complete library.

Once identified, an antibody sequence is usually transferred to a bacterial expression vector in the form of a single-domain antibody (including a polyhistidine tag for immobilized-metal affinity chromatography purification, and often a pelB leader sequence for periplasmic localization) rather than as a phage protein fusion. It is likely that the phage-display system, while effective at identifying antibodies of interest, will introduce biases in the selection process. There is no guarantee that the antibodies most easily selected by phage-display will be those with the most superior properties for use as soluble antibodies or as fusions to other proteins. Since an animal, through the process of somatic hypermutation, produces a plurality of antibody variations during a normal immune response it is an open question as to whether those identified by screening represent an average, a best fraction, or perhaps those which are merely most suited for expression in the phage display format.

In this work, we sought to employ NGS to complement traditional library construction and selection methods in order to study a larger pool of related sequences. Several steps in the traditional process of construction and panning of an sdAb library can introduce bias or result in loss of sequence diversity. For instance, enzyme bias can affect both the reverse transcription of the mRNA library from lymphocytes and the amplification of the cDNA library. Protein expression bias can influence the expression and presentation of functional sdAbs on the surface of the phage. These biases could result in the loss of library diversity and subsequent omission of potentially superior antibodies.

Typically, library screening reveals families of DNA sequences that share similar complementarity determining region (CDRs). Although these clones will bind the same epitope, they can display a range of melting temperature and often show variations in affinity [19]. It remains unknown whether these are the highest affinity and most stable antibodies within the library. It would be desirable to study similar antibodies which are present but not recovered by phage-display selection. By applying NGS to evaluate the cDNA repertoire from a previously developed sdAb library derived from a llama immunized with staphylococcal enterotoxin B (SEB) [20] and using previously identified sequences to query the entire repertoire of sequences, we sought to identify novel sdAb sequences and to characterize their stability and binding kinetics. By comparing groups of related antibodies recovered by phage display selection and by sequencing we were able to investigate the quality of the selection method.

## Materials and Methods

### Library construction

This study uses a library previously constructed and characterized [20]. Total RNA was isolated from the white blood cells collected from the blood of an SEB-immunized llama according to manufacturer’s protocol (Qiagen RNA Blood Mini Kit). A cDNA library was prepared using approximately 0.5 μg of total RNA primed by oligo dT subjected to reverse transcriptase PCR using Superscript III Reverse Transcriptase (Life Technologies). Approximately 25 ng of the resulting cDNA was then amplified in the presence of 8% DMSO for 35 cycles at a denaturing temperature 94°C for 15 sec; an annealing temperature of 56°C for 1 min 30 sec and an elongation temperature of 70°C for 45 sec using Platinum Taq DNA Polymerase (Life Technologies) and the outward primers as described previously [21]. The resulting 600 bp DNA fragments containing VH regions were then purified and served as templates (25 ng in each 50 μL reaction) to a second PCR amplification under similar PCR cycling conditions except that the annealing temperature was lowered to 54°C, the elongation step shortened to 30 sec, and the inward primers were as described previously [22]. Both outward and inward primer sequences were as described by Ghahroudi et al. [23] with the exception of the flanking restriction sequences, as shown in Table 1. The amplified DNA fragments (450–500 bp) were then separated on a 1% agarose gel running in tris-EDTA-acetate buffer and purified using the QIAquick Gel Extraction Kit followed by the QIAquick PCR Purification Kit. In our previous work a set of 10 random clones were verified by sequencing prior to screening the library by phage display. Seven of the 10 were complete antibody sequences while 3 had stop codons or frame shift mutations which corrupted the sequence.

**Table 1:** Primer Sequences.

Outward F	5’-CGCCATCAAGGTACCAGTTGA-3’
Outward R	5’-GATGTGCAGCTGCCGTCTGGRGGAGG-3’
Inward F	5'-TTATTACTCGCGGCCCAGCCGGCCATGGCCGAKGTSCAGCT-3'
Inward R	5'-GCGGCCGCGAATTCGGCCCCCGAGGCCGCTGGTTGTGGTTTTG-3'

All animal methods were carried out by Triple J Farms and were in accordance with established guidelines and regulations. The immunization protocol used in this work was approved by the Triple J Farms Institutional Animal Care and Use Committee (IACUC).

### Library sequencing

The PCR product was subjected to Next-Generation Sequencing by Eurofins Genomics. Sequences were generated with a MiSeq system using a 2x250 paired-end module. A single run was conducted which yielded 20.95 million reads and 5.24 gigabasepairs of data. The percentage of reads with Q score above 30 was 76.19% and the mean Q score was 30.59. The pair-end reads were combined using the FLASH program [24]. The result was a set of 8,573,790 individual nucleotide sequences (about 82% yield). Sequences were translated and converted to fasta format using Matlab with the BioInformatics toolbox (Mathworks). Searches within this database were carried out using BLAST (National Library of Medicine).

### Cloning and expression of sdAbs

After identification of sequences of interest from the library, the corresponding genes were synthesized by Eurofins Genomics. Synthesized genes were digested with NcoI-HF and NotI-HF restriction enzymes (New England Biolabs) and ligated into the pET22b+ expression plasmid (Novagen) using T4 DNA ligase. Upon sequence confirmation, the plasmid was transformed into Rosetta(DE3) *E*. *coli* (Novagen) to facilitate protein expression.

Protein expression was performed by growth of a 50 mL culture of the above construct in Terrific Broth in the presence of 100 μg/mL ampicillin and 35 μg/mL chloramphenicol overnight at 30°C. The following day, the culture was transferred to 500 mL of fresh broth and grown for 3 h at 30°C. Protein expression was induced with IPTG at a final concentration of 0.5 mM at 30°C for 3 h. Cells were harvested by centrifugation and the protein was harvested from the periplasm by osmotic shock and purified by immobilized metal affinity chromatography and size exclusion chromatography, as described previously [20, 25]. Gene sequences for Aa, Ac, Ad, and Ca through Cd are available in GenBank under accession numbers KU508539 through KU508545.

### Determination of the thermal stability of sdAbs

The Melting temperature was measured by circular dichroism (CD) spectroscopy. Proteins were prepared in a 1.0 cm path length quartz cuvette in 3.0 mL of water at a concentration of 20 μM. CD measurements were performed using a Jasco J-815 Spectropolarimeter equipped with a PTC-423S Peltier temperature controller. The temperature was increased from 25°C to 95°C at a rate of 2.5°C/min. Ellipticity was monitored at a wavelength of 205 nm. To monitor protein refolding, the temperature was returned to 25°C at a rate of 2.5°C/min.

The melting temperature for each of the antibodies was also determined using a thermofluor assay conducted using an Applied Biosystems Step One Real Time PCR system and Sypro Orange dye (Sigma) as described previously [20]. A total of 10 μg of each purified sdAb was added to a 20 μl final volume of PBS buffer. The Sypro Orange dye was diluted 1000-fold into each reaction solution. The temperature was increased from 25 to 99°C at a rate of 1% (about 1.2°C/min) and the fluorescence was measured via the ROX channel. All measurements were done in triplicate.

### Determination of sdAb binding kinetics

Surface Plasmon Resonance affinity and kinetics measurements were performed as described previously using the ProteOn XPR36 (Bio-Rad) [26]. Immobilization of SEB onto a GLC chips was performed in 10 mM acetate buffer pH 5.0 by standard 1-Ethyl-3-(3-dimethylaminopropyl)-carbodiimide hydrochloride coupling chemistry. Binding kinetics of each sdAb were determined at 25°C by flowing six concentrations of antibody varying from 300 to 0 nM at 100 μL/min for 90 s over the SEB-coated chip and monitoring dissociation for 600 s. Following each run, the chip was regenerated by 0.85% phosphoric acid for 36 s. Data analysis was performed with ProteOn Manager 3.1 software, corrected by subtraction of the antibody blank column as well as the interspot correction. Binding constants were determined by the manufacturer's software using the Langmuir model.

## Results

### Classification of antibodies selected by phage display

**Fig 1:**
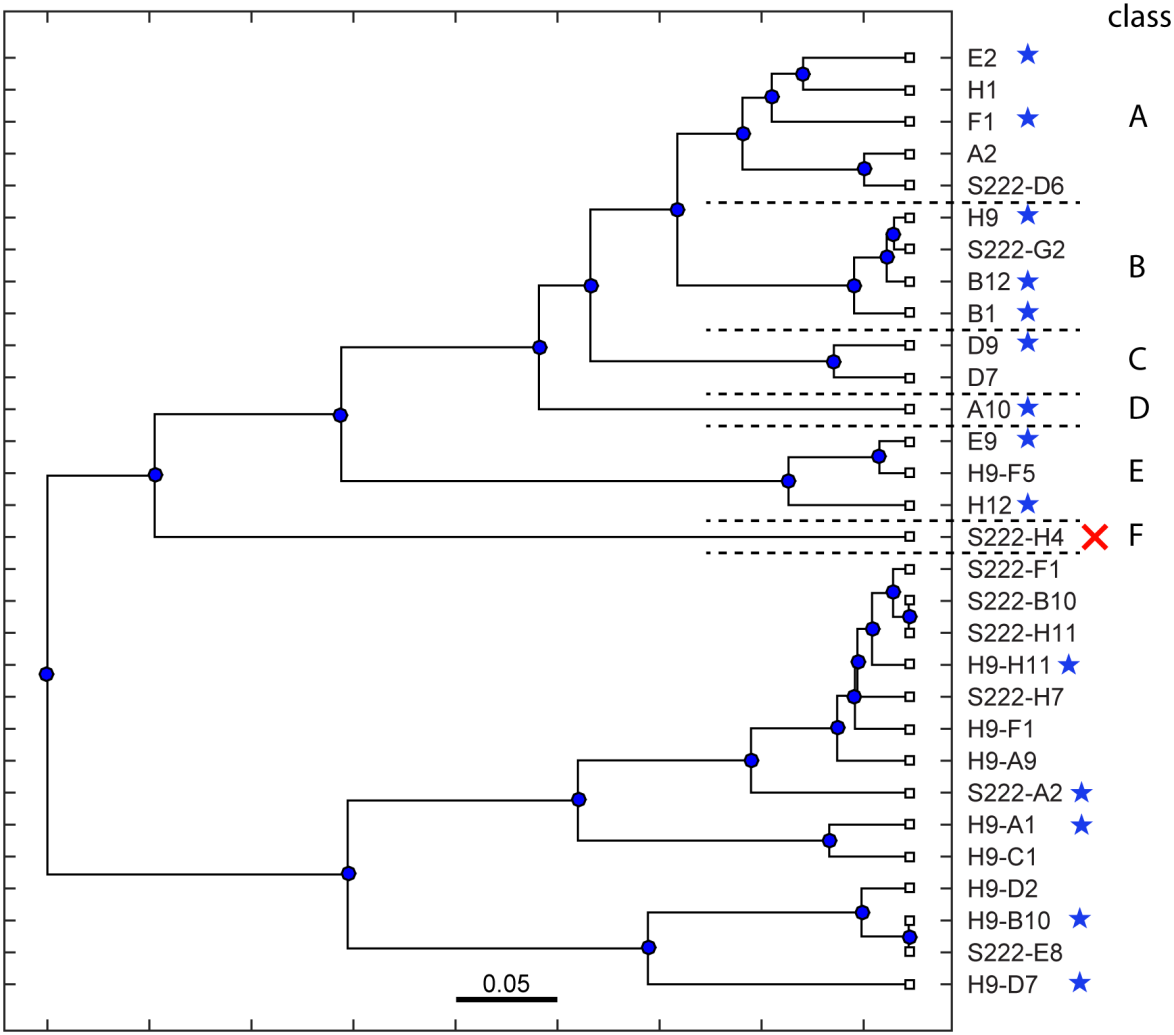
Phylogenetic tree of previously identified antibody sequences. Sequences of antibodies isolated by phage panning of the sequenced library in a previous work were grouped based on sequence similarity. A blue star indicates antibodies that were characterized in the previous work. The red X indicates an antibody that did not bind SEB. The scale bar represents a 5% difference in amino acid sequence. Single names refer to antibodies recovered by direct binding to immobilized antigen. Hyphenated names indicate those which were recovered by a sandwich method.

In prior work we constructed a phage display library of sdAbs derived from a llama immunized with inactivated SEB [20]. A cDNA library was prepared from mRNA isolated from lymphocytes of an SEB-immunized llama and primers specific for the variable domains of camelid antibodies were used to amplify the sdAb sequences. Functional antibodies were selected from this library by two methods. First, by direct binding of the phage-display library against the immobilized antigen. Of nine tested antibodies, all competed for binding toxin and thus were presumed to bind an identical or overlapping epitope. A second method was employed using a sandwich format in which the antigen was immobilized by binding via one of these newly isolated antibodies. A second set of antibodies were recovered, most of which were shown to bind to a different epitope as expected. Fig 1 shows a phylogenetic tree of the antibody sequences recovered from the phage-display library. The antibodies cluster as would be expected with those binding each epitope segregated from the other. Antibodies which were characterized by affinity, competition and melting point in our previous paper are indicated by a blue star. One, S222-H4, was shown to not bind to SEB and was presumed to be an artifact. Antibodies with hyphenated names were recovered by the second, sandwich, method. Three of those had high sequence similarity to those recovered by the first method and were presumed to bind to the first epitope.

Antibodies recovered by the direct binding method of selection were grouped into 6 classes based on sequence similarity. In Fig 1, these are indicated by dashed lines and attributed to Class A through F. These groupings will form the bases of our analysis of the sequence database.

### Construction of library sequence database

The same cDNA that was used to construct the phage display library was prepared for Next Generation Sequencing. Approximately 5 μg of the PCR product was sequenced with a MiSeq Next-Generation Sequencing system and yielded a database of 8,573,790 full-length protein sequences. In order to assess the quality of the database, three groups of 100 random entries were selected from the library and manually examined for frameshifts, internal stop codons, and gross corruption of the expected sequence. These are presumed to be RT-PCR, PCR, or sequencing artifacts, and were found to compose 37.3 +/- 2.5% of the entries. This indicates a final yield of approximately 5.4 million intact, useful sequences. This proportion is consistent with the results found by sequencing random clones to check library quality prior to screening (see Materials and Methods section).

**Fig 2:**
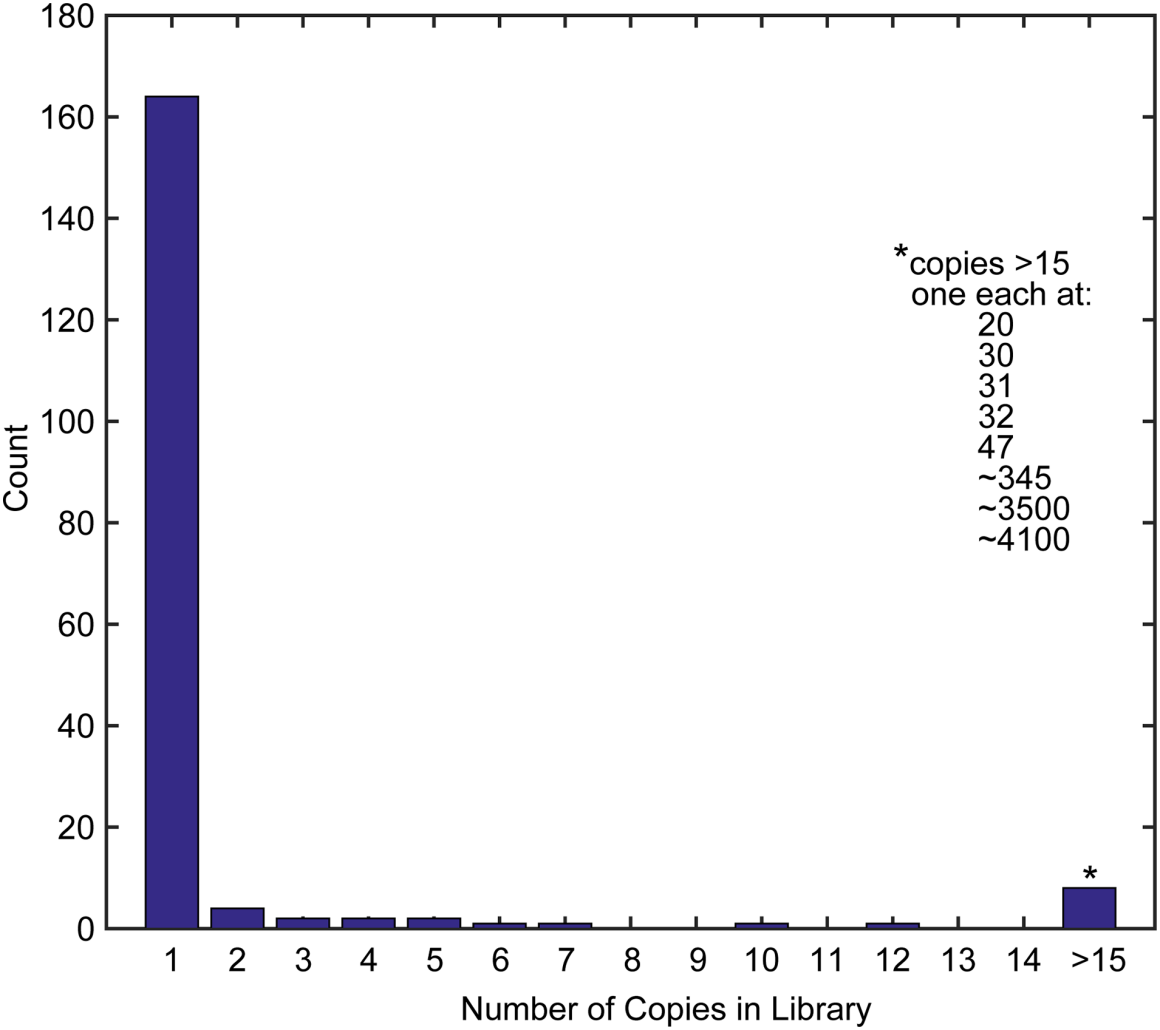
Histogram of sequence copy number in the library. Using 186 randomly-selected sequences from the library, a BLAST search was used to determine the copy number of each sequence within the library. For the 8 sequences present in greater than 15 copies the copy number of each example is listed in the inset.

In addition to calculating the total number of sequences, the copy number of sequences within the library can be determined. A total of 186 intact sequences from the three random groups were used as queries to execute BLAST searches against the full sequence library. The results were examined to determine the copy number for sequences within the library. For counts greater than 100 the copy number was estimated based in the proportion of the library covered by the first 100 matches. A large majority (88%) of the random sequences are present in the library as a single copy. The histogram in Fig 2 shows that sequences present at low copy numbers (2–12) comprise less than 10% of the library. Five sequences had greater than 15 copies and three had greater than 100 copies. This indicates that the cDNA library is highly diverse with only a small minority represented by more than one copy. It also suggests that the library is not a complete record of sequences present in the animal, and that a large number of native antibodies are not represented.

In order to test the hypothesis that Next-Generation Sequencing could be used to identify additional members of antibody classes, we selected exemplars from Class A (E2) and Class C (D9) to use as queries for BLAST searches against our sequence library.

### Identification of new class members

Our hypothesis was that a relatively large number of sequences for novel antibodies could be identified by using known sequences to search the entire library repertoire. The recovery of additional class members would allow us to determine whether those recovered by screening are the most suitable for use in immunoassays. Although specific antibodies are readily recovered by current techniques, it is not known whether these methods are optimal for production of the highest affinity or most robust reagents.

**Fig 3:**
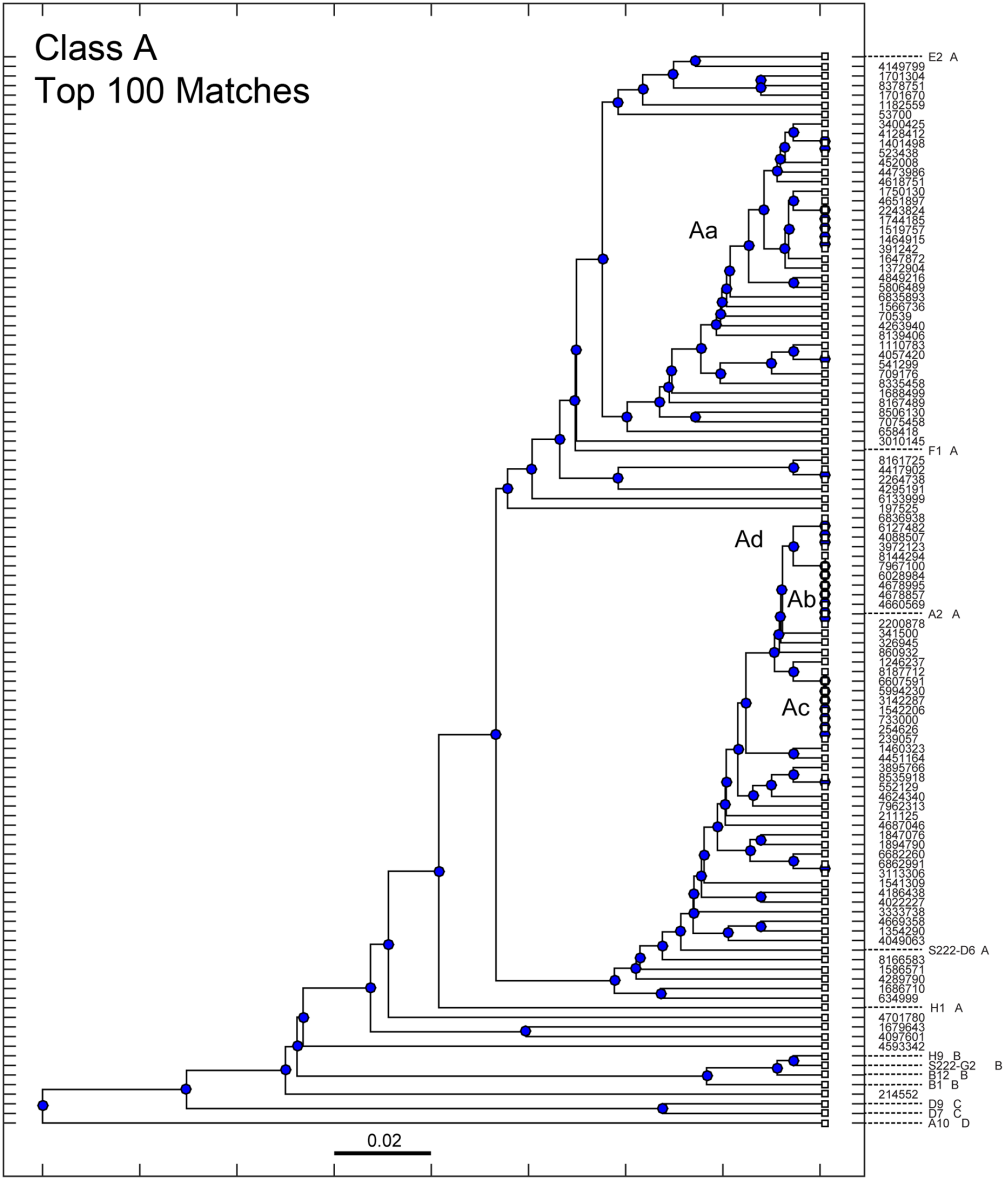
Phylogenetic tree of the top 100 sequences for Class A. Library sequences obtained in a search query using sequences from Class A were arranged in a phylogenetic tree denoting sequence similarity. The scale bar represents a 2% difference in the amino acid sequence. Previously identified sequences in Classes A through D were included, and denoted appropriately within the tree. Sequences from the library present in multiple copies are denoted as Aa, Ab, Ac, Ad.

The sequence of antibody E2 from Class A was used as a query for a BLAST search. The exact sequence of E2 was not present in the library. This is not unexpected since the high diversity of the library indicates that only a minority of native sequences present in the animal are represented in the library. Fig 3 shows a phylogenetic tree of the top 100 sequence matches to E2 and was supplemented with the members of Classes A through D. Newly-identified sequences are identified by sequence number. Known sequences are identified by name and class. Class A members are distributed throughout this collection of new sequences. Only one known sequence (A2) was found to have identical copies in the sequence library. While most new sequences were found in only a single copy, several are present in multiples and labeled Aa through Ad. These multiply-occurring antibodies were picked for further study.

**Fig 4:**
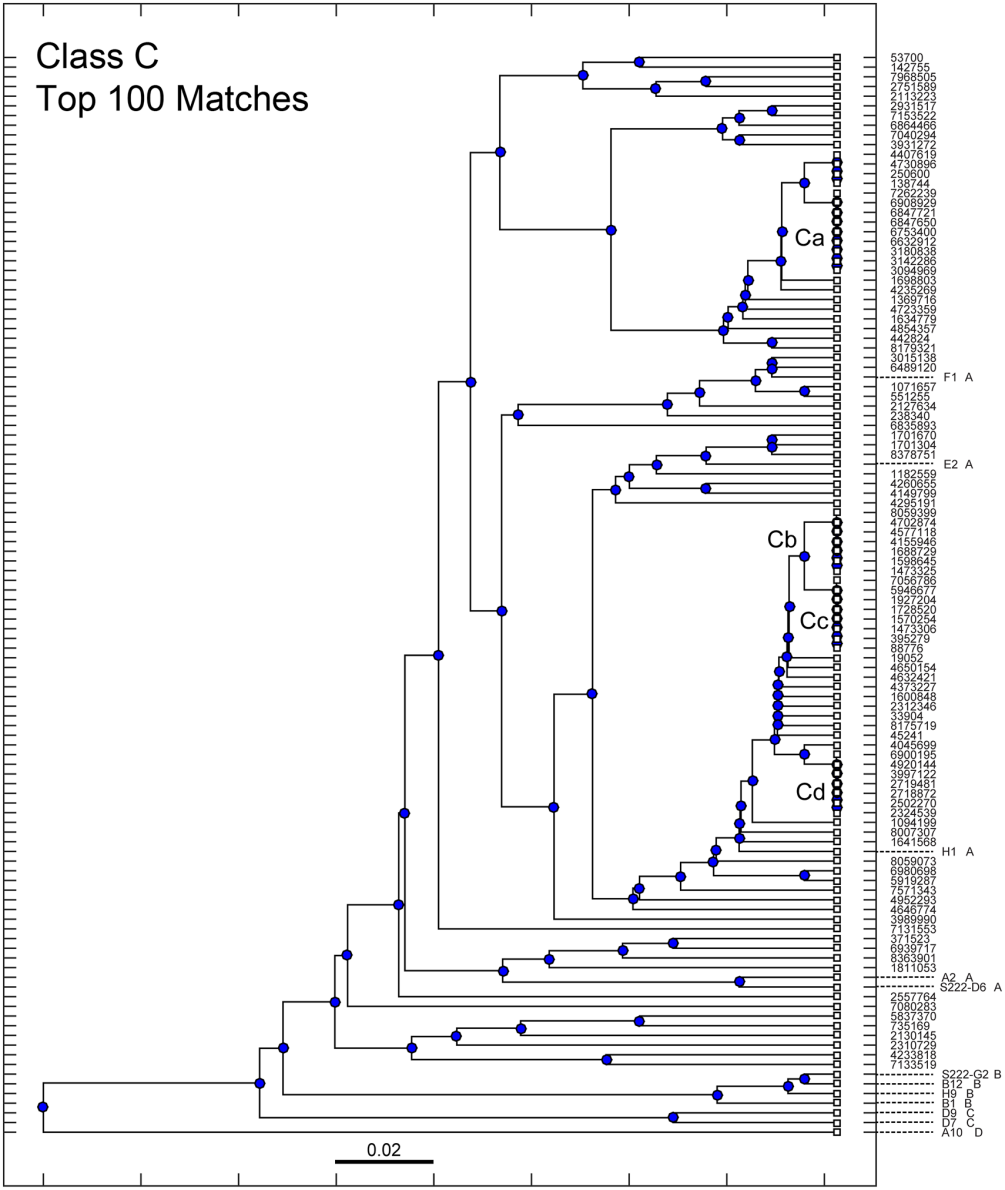
Phylogenetic tree of the top 100 sequences for Class C. Library sequences obtained in a search query using sequences from Class C were arranged in a phylogenetic tree denoting sequence similarity. The scale bar represents a 2% difference in the amino acid sequence. Previously identified sequences in Classes A through D were included and denoted appropriately within the tree. Sequences from the library present in multiple copies are denoted as Ca, Cb, Cc, Cd.

Similarly, the sequence of D9 was used as an exemplar from Class C. Fig 4 shows the top 100 matches to D9, again plotted with the known members of Classes A through D. These data are similar in that most new sequences are present in a single copy but with several clusters of sequences present at a high copy number. None of the sequences were matches to either D9 or D7, the two members of Class C. In fact all of the top 100 matches to sequence D9 were more closely related to members of Class A than to D9. This suggests that Class C is much less common than Class A in the library, and implies that this may be true also of the native antibody repertoire in the animal. However, given the nature of RT-PCR and PCR, we cannot feel assured that this result is not an artifact. Nevertheless, we also observe in this data the appearance of certain sequences with more than one copy. The sequences present as multiples are labeled as Ca through Cd.

The sequences in each class present in multiple copies were selected for further study. It is not known whether these sequences are present in higher numbers of circulating B cells in the organism, or if their presence at a higher copy number is due to PCR or sequencing artifacts. Given the high diversity of this library, it is at least plausible that these sequences represent an immunologically preferred antibody.

**Fig 5:**
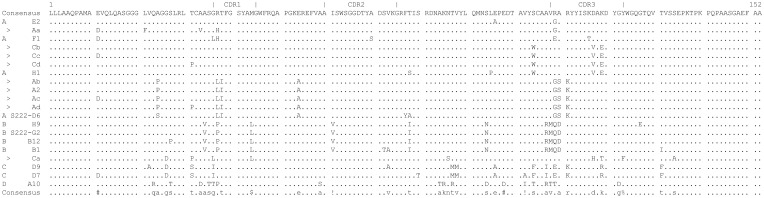
Sequences of antibodies studied in this work. Sequences are shown as a difference plot where a dot represents identity with the consensus sequence. Novel antibodies are identified by a > symbol and their two-letter abbreviations as used the in the text. Class A through D antibodies are shown for comparison and identified by name and class. Complementarity Determining Regions 1 through 3 are indicated.

A difference plot of antibody sequences showing Class A through D together with the selected new sequences is shown in Fig 5. The locations of the CDRs are indicated at the top of the figure. Sequences from our previous work are indicated by class. Novel sequences are indicated with a > symbol.

### Thermal stability of selected antibodies

In order to determine the stability of the various antibodies, bacterial expression was employed to produce antibodies Aa, Ac, Ad, and Ca through Cd (Sequence Ab was not produced because it is identical to the previously-studied antibody A2). The selected antibodies were cloned into an expression vector, produced, and purified as described in the Methods section. Yields for antibodies produced in 500 mL culture were approximately 5.0 mg with the exception of antibody Cd, which yielded 1.5 mg. Melting temperatures were determined using two different methods and are shown in Table 2. Melting temperatures determined by circular dichroism ranged from 47°C to 75°C with an average of 67°C. Melting points determined by a thermofluor assay ranged from 47°C to 73°C with an average of 64°C. In comparison the melting temperatures for the exemplars are 72°C and 65°C as measured by circular dichroism for E2 and D9 respectively [20]. Several of the antibodies newly identified by this work displayed an improved thermal stability as compared with the previously identified examples. This shows that by deep sequencing of libraries it will be possible to find superior antibodies, even though overall the range of stability recovered will be similar.

**Table 2:** Thermal Stability and Affinity Characterization of sdAbs.

Antibody	Tm, °C (CD)	Tm, °C (TFA)	k_a_ (1/Ms)	k_d_ (1/s)	K_D_ (M)
**Aa**	74	71	8.6 x 10^5^	5.3 x 10^−4^	6.3 x 10^−10^
**Ac**	75	73	4.5 x 10^5^	3.0 x 10^−4^	6.7 x 10^−10^
**Ad**	60	55	4.8 x 10^5^	4.6 x 10^−4^	9.6 x 10^−10^
**Ca**	47	47	3.2 x 10^5^	2.2 x 10^−4^	6.9 x 10^−10^
**Cb**	72	70	1.7 x 10^6^	6.1 x 10^−4^	3.6 x 10^−10^
**Cc**	73	72	8.7 x 10^5^	6.9 x 10^−4^	7.9 x 10^−10^
**Cd**	nd*	59	5.1 x 10^5^	6.0 x 10^−4^	1.2 x 10^−10^
**E2**	72	71	1.3 x 10^7^	3.5 x 10^−3^	2.7 x 10^−10^
**D9**	65	63	2.5 x 10^6^	2.8 x 10^−4^	1.1 x 10^−10^

nd*—not determined, did not produce melting curve

### Binding affinity of selected antibodies

Binding affinities and kinetics of the interaction of selected sdAbs with their antigen were determined by surface plasmon resonance (SPR) and are shown in Table 2. On-rates (k_a_) ranged from 3.2 x 10^5^ 1/Ms to 1.7 x 10^6^ 1/Ms and off-rates (k_d_) ranged from 2.2 x 10^−4^ 1/s to 6.9 x 10^−4^ 1/s. Equilibrium dissociation constants (K_D_) ranged from 1.2 x 10^−10^ M to 9.6 x 10^−10^ M. The on-rates for sdAb E2 and D9 were measured at 1.3 x 10^7^ and 2.5 x 10^6^ 1/Ms, while their off-rates are 3.5 x 10^−3^ and 2.8 x 10^−4^ 1/s, leading to K_D_ values of 2.7 and 1.1 x 10^−10^ M.

These results show that the new antibodies have affinities to SEB similar to those identified by screening with phage display. None of the tested sequences had lost the ability to bind the antigen. This is consistent with our hypothesis that sequences which occur in multiple copies are representative of antibodies circulating in the animal as a result of the immune response. While the potential of artifactual sequence alteration cannot be ruled out, many such random mutations would be expected to be detrimental to stability or affinity. The absence of such consequences gives us confidence that our procedure will have a more general utility.

### High copy number antibodies

During analysis of the sequence database, it was observed that a few sequences were present in greater than 100 copies. Three sequences were observed with estimated copy numbers between 350 and 4100 copies. A further 300 randomly-selected sequences were examined and nine additional examples were found with copy numbers between 280 and 1100. Sequences and copy numbers are shown in Table 3. These sequences were unrelated to the antibodies identified by screening via phage display. To test whether these antibodies had affinity to SEB, two were synthesized and assayed (identified by an asterisk in Table 3). No affinity to SEB was observed and so no further characterization of other high-copy number sequences was pursued.

**Table 3:** Sequences of high-copy-number antibodies.

ID	Copy Number	Sequence
697006	345	LLLAAQPAMAEVQLQASGGGFVHTGHSLRLSCECSGRALRTTAWFRQAPGQGREFVAAIRWDDAVTEFSDSAKGRFAISRGGGDNTVNLDMNNLKPDDTAVYFCAAQSPGPTPHSLSIAGEYDHWGQGTQVTVSSEPKTPKPQPAASGAEFAA
4316133	4100*	LLLAAQPAMADVQLQASGGGVVQVGESLRLSCRLFGNTFSNFAVGWFRQAPGKAREFVGNMGRSGISTYYDDSVKGRFTIAKDNADNLAVLIMSMLKPADTGTYYCAAGPQPYAREAGYDYWGQGAQVTVSSEPKTPKPQPAASGAEFAA
237221	3500	LLLAAQPAMAEVQLQASGGGVVQVGESLRLSCRLFGNTFSNFAVGWFRQAPGKAREFVGNMGRSGISTYYDDSVKGRFTIAKDNADNLAVLIMSMLKPADTGTYYCAAGPQPYAREAGYDYWGQGAQVTVSSEPKTPKPQPAASGAEFAA
8563088	1100*	LLLAAQPAMADVQLQASGGGSVQAGGSLRLSCAASISLSRLHPLSWYRQTPGNQRELVAVITLGGSTTYADSVKGRFTISRDNANKIFDLEMRNLKPEDTAVYYCSAAGTYWGQGTQVTVSAEPKTPKPQPAASGAEFAA
8520137	280	LLLAAQPAMADVQLQASGGGFVEAGGSLSLSCTMSGFNMNDYCMAWFRRAPGKGRKGVASIGKSYGRTYYEESVKGRFTISMDKAKRTVYLQMSGLKPDDTAVYYCTVHRDQDGEECDLKYEYYDHWGKGTQVSVSSEPKTPKPQPAASGAEFAA
8569230	460	LLLAAQPAMADVQLQASGGGLVQPGGSTKLSCTASGEISEIVRYDWYRLAPGTERDWDTSQRDWVATAATGGAINYADSVKGRFTIALIRGDNQDTVHLQMGNLTPADTAVYFCSARSRWYDDPEYWGQGTQVTVASEPKTPKPQPAASGAEFAA
8559527	500	LLLAAQPAMADVQLQASGGGLVQPGHSLTISCVASGSAIKPYTMAWFRQAPGKEREFVVAQKRIGGNVYSSDYAESVKGRFSISRNNAKNTVTLEMNSLKSEDTAVYTCAAAESGRLPLTDPHQYPYWGQGTQVTVSSEPKTPKPQPAASGAEFAA
8573639	480	LLLAAQPAMADVQLQASGGGSVQAGANLRLSCVVSGLTYDTTGVVWFRQAPGKERQFVAGLRWDGGSTYYADSVQGRFDISKDNANNTVYLQMNNLESEDTAVYYCAADNVLTSAAYARADMYDYWGQGTQVTVSPEPKTPKPQPAASGAEFAA
8532657	380	LLLAAQPAMADVQLQASGGGALQPGGSLRLSCVFSGRYSMRDYAMGWFRQAPGKEREIVAAISRNHGRTFYQDSVKGRFTISRDDFKSTLYLQMNDVKPEDTAMYYCAARNEMANRGSREYFTAASLYGYWGQGTQVTVSSREEPKTPKPQPAASGAEFAA
8572659	280	LLLAAQPAMAEVQLQASGGGLVQAGDSLRISCTASGRTLNGGPMSWFRRVPGAERDFVAGISRSGGQTAYADFAKGRFIISIDNAENTVYLQMNSLKPEDTAVYYCAAKQRYRDYVGRRISEYDYWGQGTQVTVSSEPKTPKPQPAASGAEFAA
8543759	480	LLLAAQPAMAEVQLQASGGGRVQPGGSLRLSCTVPRTFYRPATIAWYRRPSEKEREWVASITPGGLAKYADAVMGRFTISRDDAENTVYLQMKGLEPEDTAVYYCKVETYGLWGRGTQVTVDSEPKTPKPQPAASGAEFAA
8570829	670	LLLAAQPAMADVQLQASGGGLVQAGDSLRISCTASGRTLNGGPMSWFRRVPGAERDFVAGISRSGGQTAYADFAKGRFIISIDNAENTVYLQMNSLKPEDTAVYYCAAKQRYRDYVGRRISEYDYWGQGTQVTVSSEPKTPKPQPAASGAEFAA

* These sequences tested for affinity to SEB.

## Discussion

The purpose of this study was to examine the hypothesis that deep sequencing of a library could reveal additional functional antibodies belonging to classes identified by traditional phage-display selection. There is no *a priori* guarantee that the antibodies selected during library panning as phage protein fusions will be the best antibodies for use in *in vitro* assays. It is reasonable to assume that a certain percentage of high quality antibodies will be lost due to poor performance in the screening protocol. This could occur as a result of poor production of the antibody fragment as a fusion to the phage coat protein, improper folding of the antibody fragment, or unforeseen selective bias against certain antibody sequences in *E*. *coli*. Since the cost of Next Generation Sequencing has become relatively modest, it is possible to recover the sequences of many, if not all, additional members of an antibody class identified by screening. This study is facilitated by the fact that sdAbs are very small, consisting of a functional binding element with a single chain of less than 160 amino acids. Thus sdAbs are capable of being fully sequenced by a MiSeq device.

To investigate this, we conducted a new PCR amplification of a cDNA repertoire composed of sequences for antibodies against SEB that had been used in our previous study [20]. This allowed for a re-sampling of the total available sequences rather than limiting ourselves to any potential bias caused by the construction of the phage display library. The resulting DNA library was then subjected to NGS. Over 8.5 million sequences were obtained, with over 5.4 million sequences representing complete, intact sequences.

Examination of this sequence library yielded a number of interesting observations. First, it was apparent that the library was of exceptional diversity; over 80% of sequences appear to be present at a single copy number. Another 10% of sequences are present at a low copy number (n < 12) and only a handful of sequences were present at very high copy numbers. It is unclear whether sequences present at high copy numbers represent antibodies present in a greater number of circulating immune cells in the animal or if they are an artifact of either the PCR employed in library preparation or the sequencing procedure itself. Antibodies corresponding to two of these high-copy number sequences were produced and did not show affinity to SEB. Therefore, if these sequences do represent an immunologically-relevant antibody, they likely recognize a different antigen. Database searches did not return any matches to these sequences. Further work with other repertoire libraries may allow us to say more about the significance of these high copy number examples.

Many sequences were excluded from analysis when the paired-ends failed to be connected by the FLASH algorithm. This is likely due to the deterioration of sequence quality with read length. The MiSeq 2X250 paired-end module reads 250 basepairs from each end and is therefore capable of producing overlaps for sequences of somewhat less than 500 basepairs. The PCR product used for this study was at the upper range for this application and will have produced overlaps of only 20–40 basepairs. This short overlap combined with lower sequence reliability is probably responsible for many of the failed pairings. The MiSeq platform now has available a 2X300 paired-end module which may be useful for future work.

After the database was compiled, the sequences were searched using candidate antibodies obtained previously via a standard phage-display selection method. Antibodies present at a multiple copy number and deemed similar to the sequence of known SEB binding antibodies were produced and characterized in terms of affinity and stability. All seven of the characterized antibodies exhibited binding to SEB with sub-nM affinities, showing comparative affinities with those obtained through panning [20]. Average melting temperature for those identified by phage-display was 65°C, while the average for those identified by sequence similarity was 67°C, showing good agreement. Several of the sdAbs identified by library sequencing showed superior melting temperature compared to those originally identified by traditional selection. The affinity of the novel antibodies was slightly inferior to those identified by selection, but were of the same scale.

These results demonstrate that by interrogating the vast ensemble of sequences present in a library using sequences of known binders, it is possible to quickly identify numerous new antibodies with a comparable range of affinity and stability characteristics. With a high-diversity library it may often be possible to recover superior antibodies by this method. Moreover, with the decreasing cost of gene synthesis services, it is possible to identify, construct, produce, and characterize a higher number of novel antigen-binding candidates more rapidly than would be achievable using a traditional phage-display screening protocol. Our results suggest that those antibodies identified by screening are not necessarily those present in the greatest number in the B cell mRNA, in the cDNA library, or in the organism itself. In this instance our library was of high diversity with only a small minority of high copy number duplications. If those sequences represent B cell lines most amplified by the immune response then it may well be that phage-display screening often fails to identify the most immunologically active antibodies.

One nagging question that remains, and is common to all techniques involving NGS, is whether the antibodies discovered occur in the animal, or are they artifacts due to PCR or sequencing errors. It is well understood that in mass sequencing of highly diverse genes it will be impossible to distinguish fact from artifact [14]. This can be of great concern for those studying the underlying immune response of animals. In our case, however, the problem is less pressing. When the focus is on the acquisition of functional antibodies for biotechnical application the importance is on affinity and stability, rather than on the reproduction of a native antibody. In any case, the recent development of a new technique for bar-coding of cDNA sequences may be useful in the future for control and analysis of sequencing and PCR artifacts [13].

The use of NGS may be of use in cases where despite the researchers' best efforts an antibody library fails to meet expectations. Screening for some targets is problematical, some antibodies recovered have regrettably low affinity, and sometimes discovery of novel antibodies is frustrated when a promising phage-display fusion fails to yield equally high affinity when produced as a free antibody. In these and other cases it will be useful to conduct NGS in order to examine the plurality of related antibodies.
